# [1-Phenyl-2-(4-pyrid­yl)ethyl­idene]hydrazine

**DOI:** 10.1107/S1600536809014330

**Published:** 2009-04-22

**Authors:** Si-Ping Tang

**Affiliations:** a Department of Chemistry and Material Science, Hengyang Normal University, Hengyang, Hunan 421008, People’s Republic of China

## Abstract

The title compound, C_13_H_13_N_3_, is non-planar, with the pyridine and phenyl rings inclined at an angle of 80.7 (3)°. The central ethyl­idenehydrazine atoms lie in a plane [mean deviation = 0.013 (1) Å], which forms dihedral angles of 88.5 (1) and 9.4 (1)° with the pyridine and phenyl rings, respectively. In the crystal structure, mol­ecules are linked by inter­molecular N—H⋯N hydrogen bonds into infinite chains propagating along the *b* axis.

## Related literature

For related structures of hydrazine derivatives, see: De *et al.* (2006 ▶); Patra & Goldberg (2003 ▶).
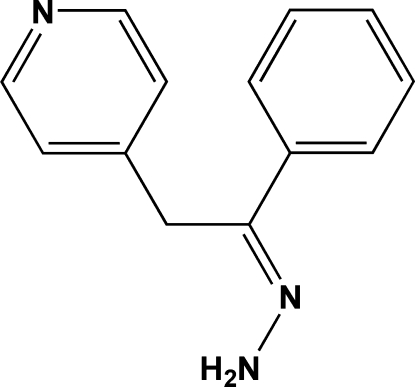


## Experimental

### 

#### Crystal data

C_13_H_13_N_3_
*M* *_r_* = 211.26
Orthorhombic, 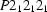

*a* = 5.7428 (6) Å
*b* = 10.8751 (11) Å
*c* = 17.6358 (18) Å
*V* = 1101.4 (2) Å^3^
*Z* = 4
Mo *K*α radiation
μ = 0.08 mm^−1^
*T* = 295 K
0.30 × 0.22 × 0.15 mm

#### Data collection

Bruker SMART APEX area-detector diffractometer
Absorption correction: multi-scan (*SADABS*; Sheldrick, 1996 ▶) *T*_min_ = 0.961, *T*_max_ = 0.982
5694 measured reflections
1266 independent reflections
1117 reflections with *I* > 2σ(*I*)
*R*_int_ = 0.026

#### Refinement

*R*[*F* ^2^ > 2σ(*F* ^2^)] = 0.037
*wR*(*F* ^2^) = 0.105
*S* = 1.04
1266 reflections
145 parameters
H-atom parameters constrained
Δρ_max_ = 0.11 e Å^−3^
Δρ_min_ = −0.13 e Å^−3^

### 

Data collection: *SMART* (Bruker, 2002 ▶); cell refinement: *SAINT* (Bruker, 2002 ▶); data reduction: *SAINT*; program(s) used to solve structure: *SHELXS97* (Sheldrick, 2008 ▶); program(s) used to refine structure: *SHELXL97* (Sheldrick, 2008 ▶); molecular graphics: *SHELXTL* (Sheldrick, 2008 ▶); software used to prepare material for publication: *SHELXTL*.

## Supplementary Material

Crystal structure: contains datablocks I, global. DOI: 10.1107/S1600536809014330/sj2620sup1.cif

Structure factors: contains datablocks I. DOI: 10.1107/S1600536809014330/sj2620Isup2.hkl

Additional supplementary materials:  crystallographic information; 3D view; checkCIF report

## Figures and Tables

**Table 1:** Hydrogen-bond geometry (Å, °)

*D*—H⋯*A*	*D*—H	H⋯*A*	*D*⋯*A*	*D*—H⋯*A*
N3—H1N⋯N1^i^	0.86	2.24	3.040 (3)	154

Symmetry code: (i) 
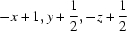
.

**Fig. 1.**
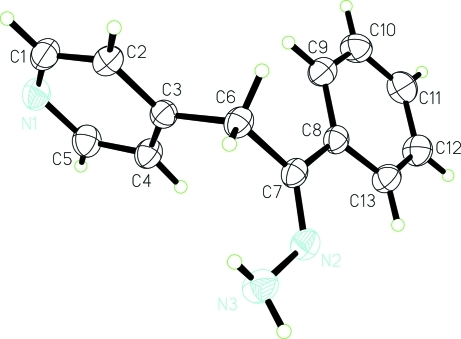
The title molecule with displacement ellipsoids drawn at the 30% probability level, and H atoms as spheres of arbitrary radius.

**Fig. 2.**
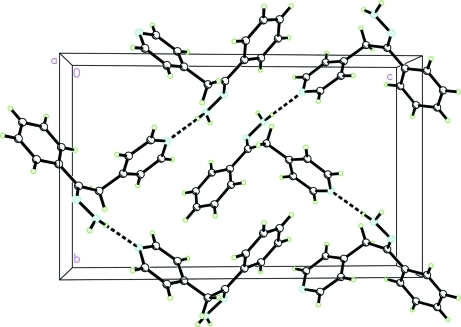
Packing diagram of the title structure showing the N—H···.N hydrogen bonding interactions as dashed lines.

## supplementary crystallographic information

### Comment

The chemical properties of hydrazine derivatives with various substitution patterns have been investigated extensively, because of their ability to bind to transition metal ions or to form unusual organic helical chains through intermolecular hydrogen bonds (De *et al.*, 2006; Patra & Goldberg, 2003). A new hydrazine derivative has been synthesized and its crystal structure is reported here, Fig. 1.

The whole molecule is nonplanar with a dihedral angle of 80.7 (3)° between the pyridine and phenyl ring. However, the central C6/C7/N2/N3 motifs are planar with the mean deviation from the plane of 0.013 (1) Å, which also generates dihedral angles of 88.5 (1)° and 9.4 (1)° with the pyridine and phenyl rings, respectively. The N2 atom forms an intramolecular C—H···N hydrogen bond with phenyl ring H13 atoms.

The crystal packing (Fig. 2) shows the amino group acts as a donor to form an intermolecular N—H···N hydrogen bond towards pyridine N atom forming infinite chains parallel to the *b* axis.

### Experimental

Benzoyl chloride (4.85 g, 34.5 mmol) was added to a solution of 4-methylpyridine (4.14 g, 44.5 mmol) in chloroform (20 ml) over 1 h at room temperature. The resulting solution was stirred for 5 h and the solvent was evaporated under vacuum to give an orange precipitate, which were triturated with toluene (20 ml) to obtain an orange solution. Then hydrazine hydrate (4 ml, 80%, 66 mmol) was added to this solution and stirred for 10 h. The solvent was removed under reduced pressure and the residue was recrystallized from dichloromethane to give light-yellow prism-like crystals of the title compound. Yield: 0.82 g (11%).

### Refinement

The carbon-bound H atoms were placed at calculated positions (C—H = 0.93 Å or 0.97 Å) and refined as riding, with *U*(H) = 1.2*U*_eq_(C). The amine H atoms were located in a difference Fourier map and allowed to ride on the N atom with N—H = 0.86 Å, *U*_iso_ = 1.2*U*_eq_(N). In the absence of significant anomalous dispersion effects, Freidel pairs were merged.

### Crystal data

**Table:**

C_13_H_13_N_3_	*F*(000) = 448
*M**_r_* = 211.26	*D*_x_ = 1.274 Mg m^−^^3^
Orthorhombic, *P*2_1_2_1_2_1_	Mo *K*α radiation, λ = 0.71073 Å
Hall symbol: P 2ac 2ab	Cell parameters from 1854 reflections
*a* = 5.7428 (6) Å	θ = 2.3–22.4°
*b* = 10.8751 (11) Å	µ = 0.08 mm^−^^1^
*c* = 17.6358 (18) Å	*T* = 295 K
*V* = 1101.4 (2) Å^3^	Prism, light yellow
*Z* = 4	0.30 × 0.22 × 0.15 mm

### Data collection

**Table:**

Bruker SMART APEX area-detector diffractometer	1266 independent reflections
Radiation source: fine-focus sealed tube	1117 reflections with *I* > 2σ(*I*)
graphite	*R*_int_ = 0.026
φ and ω scans	θ_max_ = 26.0°, θ_min_ = 2.2°
Absorption correction: multi-scan (*SADABS*; Sheldrick, 1996)	*h* = −6→7
*T*_min_ = 0.961, *T*_max_ = 0.982	*k* = −12→13
5694 measured reflections	*l* = −21→20

### Refinement

**Table:**

Refinement on *F*^2^	Primary atom site location: structure-invariant direct methods
Least-squares matrix: full	Secondary atom site location: difference Fourier map
*R*[*F*^2^ > 2σ(*F*^2^)] = 0.037	Hydrogen site location: inferred from neighbouring sites
*wR*(*F*^2^) = 0.105	H-atom parameters constrained
*S* = 1.04	*w* = 1/[σ^2^(*F*_o_^2^) + (0.0614*P*)^2^ + 0.1001*P*] where *P* = (*F*_o_^2^ + 2*F*_c_^2^)/3
1266 reflections	(Δ/σ)_max_ < 0.001
145 parameters	Δρ_max_ = 0.11 e Å^−^^3^
0 restraints	Δρ_min_ = −0.13 e Å^−^^3^

### Special details

**Table:**

Geometry. All esds (except the esd in the dihedral angle between two l.s. planes) are estimated using the full covariance matrix. The cell esds are taken into account individually in the estimation of esds in distances, angles and torsion angles; correlations between esds in cell parameters are only used when they are defined by crystal symmetry. An approximate (isotropic) treatment of cell esds is used for estimating esds involving l.s. planes.
Refinement. Refinement of F^2^ against ALL reflections. The weighted R-factor wR and goodness of fit S are based on F^2^, conventional R-factors R are based on F, with F set to zero for negative F^2^. The threshold expression of F^2^ > 2sigma(F^2^) is used only for calculating R-factors(gt) etc. and is not relevant to the choice of reflections for refinement. R-factors based on F^2^ are statistically about twice as large as those based on F, and R- factors based on ALL data will be even larger.

### Fractional atomic coordinates and isotropic or equivalent isotropic displacement parameters (Å^2^)

**Table:**

	*x*	*y*	*z*	*U*_iso_*/*U*_eq_	
N1	0.6002 (4)	0.87928 (18)	0.21104 (10)	0.0622 (6)	
N2	0.1584 (3)	1.14748 (17)	0.45942 (10)	0.0530 (5)	
N3	0.1184 (4)	1.23449 (18)	0.40448 (10)	0.0640 (6)	
H1N	0.2341	1.2665	0.3807	0.077*	
H2N	0.0076	1.2819	0.4194	0.077*	
C1	0.7655 (5)	0.9611 (2)	0.22847 (12)	0.0602 (6)	
H1	0.8945	0.9665	0.1968	0.072*	
C2	0.7567 (4)	1.0378 (2)	0.29020 (11)	0.0542 (6)	
H2	0.8782	1.0921	0.2998	0.065*	
C3	0.5646 (4)	1.03360 (19)	0.33835 (10)	0.0454 (5)	
C4	0.3919 (4)	0.9510 (2)	0.31979 (12)	0.0533 (6)	
H4	0.2587	0.9452	0.3496	0.064*	
C5	0.4169 (4)	0.8766 (2)	0.25667 (12)	0.0619 (6)	
H5	0.2981	0.8213	0.2456	0.074*	
C6	0.5549 (4)	1.11513 (19)	0.40763 (11)	0.0499 (5)	
H6A	0.5458	1.2001	0.3912	0.060*	
H6B	0.6989	1.1055	0.4358	0.060*	
C7	0.3529 (4)	1.08934 (19)	0.46036 (11)	0.0459 (5)	
C8	0.3761 (4)	0.98845 (19)	0.51709 (11)	0.0469 (5)	
C9	0.5659 (4)	0.9093 (2)	0.51678 (13)	0.0591 (6)	
H9	0.6845	0.9212	0.4816	0.071*	
C10	0.5815 (5)	0.8131 (2)	0.56787 (14)	0.0678 (7)	
H10	0.7088	0.7603	0.5665	0.081*	
C11	0.4093 (5)	0.7957 (2)	0.62054 (13)	0.0682 (7)	
H11	0.4197	0.7312	0.6550	0.082*	
C12	0.2215 (5)	0.8737 (2)	0.62229 (13)	0.0655 (7)	
H12	0.1051	0.8621	0.6583	0.079*	
C13	0.2038 (4)	0.9687 (2)	0.57141 (11)	0.0565 (6)	
H13	0.0752	1.0205	0.5732	0.068*	

### Atomic displacement parameters (Å^2^)

**Table:**

	*U*^11^	*U*^22^	*U*^33^	*U*^12^	*U*^13^	*U*^23^
N1	0.0694 (14)	0.0660 (12)	0.0512 (10)	0.0081 (11)	0.0022 (10)	−0.0091 (9)
N2	0.0551 (11)	0.0526 (10)	0.0512 (9)	0.0007 (10)	0.0080 (8)	0.0013 (8)
N3	0.0611 (13)	0.0627 (12)	0.0683 (11)	0.0049 (12)	0.0136 (10)	0.0125 (10)
C1	0.0592 (14)	0.0672 (15)	0.0544 (12)	0.0056 (13)	0.0122 (11)	−0.0001 (12)
C2	0.0489 (12)	0.0587 (14)	0.0549 (11)	0.0007 (11)	0.0078 (10)	−0.0002 (10)
C3	0.0481 (11)	0.0444 (10)	0.0438 (9)	0.0030 (9)	0.0022 (9)	0.0036 (8)
C4	0.0509 (12)	0.0570 (13)	0.0520 (11)	−0.0054 (11)	0.0076 (10)	−0.0032 (10)
C5	0.0666 (15)	0.0620 (14)	0.0571 (12)	−0.0071 (13)	−0.0007 (12)	−0.0081 (11)
C6	0.0482 (11)	0.0501 (12)	0.0513 (11)	−0.0052 (10)	0.0064 (9)	−0.0052 (9)
C7	0.0463 (11)	0.0457 (11)	0.0456 (10)	−0.0041 (10)	0.0051 (9)	−0.0089 (9)
C8	0.0481 (12)	0.0480 (11)	0.0448 (9)	−0.0043 (10)	0.0020 (9)	−0.0077 (8)
C9	0.0553 (13)	0.0653 (13)	0.0569 (12)	0.0047 (12)	0.0051 (11)	0.0008 (11)
C10	0.0663 (16)	0.0636 (15)	0.0735 (15)	0.0116 (14)	−0.0056 (14)	0.0030 (12)
C11	0.0794 (19)	0.0603 (14)	0.0648 (14)	−0.0024 (14)	−0.0045 (13)	0.0112 (11)
C12	0.0658 (16)	0.0688 (16)	0.0619 (13)	−0.0056 (14)	0.0096 (12)	0.0091 (12)
C13	0.0535 (13)	0.0593 (14)	0.0568 (11)	0.0026 (12)	0.0086 (11)	0.0022 (11)

### Geometric parameters (Å, °)

**Table:**

N1—C5	1.325 (3)	C6—C7	1.513 (3)
N1—C1	1.337 (3)	C6—H6A	0.9700
N2—C7	1.283 (3)	C6—H6B	0.9700
N2—N3	1.374 (2)	C7—C8	1.491 (3)
N3—H1N	0.8600	C8—C9	1.389 (3)
N3—H2N	0.8600	C8—C13	1.394 (3)
C1—C2	1.372 (3)	C9—C10	1.383 (3)
C1—H1	0.9300	C9—H9	0.9300
C2—C3	1.393 (3)	C10—C11	1.370 (4)
C2—H2	0.9300	C10—H10	0.9300
C3—C4	1.378 (3)	C11—C12	1.373 (4)
C3—C6	1.511 (3)	C11—H11	0.9300
C4—C5	1.384 (3)	C12—C13	1.372 (3)
C4—H4	0.9300	C12—H12	0.9300
C5—H5	0.9300	C13—H13	0.9300
			
C5—N1—C1	116.04 (19)	C7—C6—H6B	108.6
C7—N2—N3	119.61 (19)	H6A—C6—H6B	107.6
N2—N3—H1N	119.6	N2—C7—C8	116.69 (18)
N2—N3—H2N	108.8	N2—C7—C6	124.60 (19)
H1N—N3—H2N	118.5	C8—C7—C6	118.71 (19)
N1—C1—C2	124.1 (2)	C9—C8—C13	117.7 (2)
N1—C1—H1	117.9	C9—C8—C7	121.58 (18)
C2—C1—H1	117.9	C13—C8—C7	120.7 (2)
C1—C2—C3	119.5 (2)	C10—C9—C8	121.1 (2)
C1—C2—H2	120.2	C10—C9—H9	119.4
C3—C2—H2	120.2	C8—C9—H9	119.4
C4—C3—C2	116.52 (18)	C11—C10—C9	120.0 (2)
C4—C3—C6	123.25 (18)	C11—C10—H10	120.0
C2—C3—C6	120.21 (19)	C9—C10—H10	120.0
C3—C4—C5	119.8 (2)	C10—C11—C12	119.8 (2)
C3—C4—H4	120.1	C10—C11—H11	120.1
C5—C4—H4	120.1	C12—C11—H11	120.1
N1—C5—C4	123.9 (2)	C13—C12—C11	120.6 (2)
N1—C5—H5	118.0	C13—C12—H12	119.7
C4—C5—H5	118.0	C11—C12—H12	119.7
C3—C6—C7	114.62 (17)	C12—C13—C8	120.9 (2)
C3—C6—H6A	108.6	C12—C13—H13	119.6
C7—C6—H6A	108.6	C8—C13—H13	119.6
C3—C6—H6B	108.6		
			
C5—N1—C1—C2	1.5 (3)	C3—C6—C7—C8	83.3 (2)
N1—C1—C2—C3	−0.9 (3)	N2—C7—C8—C9	171.57 (19)
C1—C2—C3—C4	−0.3 (3)	C6—C7—C8—C9	−7.5 (3)
C1—C2—C3—C6	178.50 (18)	N2—C7—C8—C13	−6.9 (3)
C2—C3—C4—C5	0.9 (3)	C6—C7—C8—C13	174.03 (18)
C6—C3—C4—C5	−177.9 (2)	C13—C8—C9—C10	0.9 (3)
C1—N1—C5—C4	−0.9 (3)	C7—C8—C9—C10	−177.5 (2)
C3—C4—C5—N1	−0.3 (3)	C8—C9—C10—C11	−0.8 (4)
C4—C3—C6—C7	6.5 (3)	C9—C10—C11—C12	0.2 (4)
C2—C3—C6—C7	−172.19 (19)	C10—C11—C12—C13	0.4 (4)
N3—N2—C7—C8	−174.61 (17)	C11—C12—C13—C8	−0.3 (3)
N3—N2—C7—C6	4.4 (3)	C9—C8—C13—C12	−0.4 (3)
C3—C6—C7—N2	−95.7 (2)	C7—C8—C13—C12	178.1 (2)

### Hydrogen-bond geometry (Å, °)

**Table:**

*D*—H···*A*	*D*—H	H···*A*	*D*···*A*	*D*—H···*A*
N3—H1N···N1^i^	0.86	2.24	3.040 (3)	154

Symmetry codes: (i) −*x*+1, *y*+1/2, −*z*+1/2.
